# Structural evolutions and hereditary characteristics of icosahedral nano-clusters formed in Mg_70_Zn_30_ alloys during rapid solidification processes

**DOI:** 10.1038/srep43111

**Published:** 2017-02-23

**Authors:** Yong-Chao Liang, Rang-Su Liu, Quan Xie, Ze-An Tian, Yun-Fei Mo, Hai-Tao Zhang, Hai-Rong Liu, Zhao-Yang Hou, Li-Li Zhou, Ping Peng

**Affiliations:** 1 School of Physics and Microelectronics Science, Hunan University, Changsha, 410082, China; 2 College of Big Data and Information Engineering, Guizhou University, Huaxi District, Guiyang, 550025, China; 3 Department of electronic and communication engineering, Changsha University, Changsha, 410003, China; 4 College of Materials Science and Engineering, Hunan University, Changsha, 410082, China; 5 Department of Applied Physics, Changan University, Xi’an, 710064, China; 6 Department of Information Engineering, Gannan Medical University, Ganzhou, 341000, China

## Abstract

To investigate the structural evolution and hereditary mechanism of icosahedral nano-clusters formed during rapid solidification, a molecular dynamics (MD) simulation study has been performed for a system consisting of 10^7^ atoms of liquid Mg_70_Zn_30_ alloy. Adopting Honeycutt-Anderson (HA) bond-type index method and cluster type index method (CTIM-3) to analyse the microstructures in the system it is found that for all the nano-clusters including 2~8 icosahedral clusters in the system, there are 62 kinds of geometrical structures, and those can be classified, by the configurations of the central atoms of basic clusters they contained, into four types: chain-like, triangle-tailed, quadrilateral-tailed and pyramidal-tailed. The evolution of icosahedral nano-clusters can be conducted by perfect heredity and replacement heredity, and the perfect heredity emerges when temperature is slightly less than *T*_*m*_ then increase rapidly and far exceeds the replacement heredity at *T*_*g*_; while for the replacement heredity, there are three major modes: replaced by triangle (3-atoms), quadrangle (4-atoms) and pentagonal pyramid (6-atoms), rather than by single atom step by step during rapid solidification processes.

It is well known that local atomic structures play a crucial role in the glass-forming mechanisms and the unique properties of metallic glasses and many works have been performed on the characterization of such structures at various scales. With excellent rotational symmetry, the icosahedral (ICO) clusters have been attracting special attentions. Shechtman *et al*.^1^ firstly discovered the ICO symmetry in a metastable solid formed from melt Al–Mn alloy, by analyzing the curious diffraction pattern which has no crystallographic symmetries. With the development of the experimental techniques, the icosahedral cluster can be observed directly by the high-resolution electron microscopic technique^2^. And the systematic mapping of icosahedral short-range order can be obtained by analyzing the angular correlations in scanning electron nano-diffraction patterns^3^. The ICO clusters not only affect the thermodynamics properties of molten alloys^4^,^5^, but also exhibit their excellent stability^6^,^7^,^8^. Riccardo *et al*.^9^,^10^ analyzed the atomic stress and pressure in the icosahedral clusters, since the equilibration of local pressure is an important driving force for stabilizing structures. Polyicosahedral structures have been found also to be of special stability for free nanoalloys^11^,^12^. The glass forming abilities, thermal stability and crystallization behavior of the metallic glasses are correlated with the ICO short range order^13^. Although the five-fold icosahedra are unable to build a structure with three dimensional periodicity, they can link together to form extended clusters^14^; the length scales of such extended clusters are typically beyond the diameter of the third coordination shell and often extend to nanometer range^15^,^16^,^17^,^18^. Compared to other clusters the ICO clusters in the model systems display a strong spatial correlation, suggesting a string-like ICO medium-range atomic order^19^,^20^,^21^,^22^. The co-existence of icosahedral and chain-like structures might be a key ingredient for understanding the mechanical properties of Ni_40_Ta_60_ metallic glasses^23^. And the size-distribution of these ICO structures usually display a magic number sequence^24^,^25^.

At present, most works are still restricted in a small-scale system due to the limitation of computational capacity. Thus it is difficult to reveal the details of ICO nano-clusters with larger scale. For example, the possible geometry configurations and the magic number sequences of ICO nano- clusters including 2~8 ICO clusters are still not clear^26^. Though the evolution of ICO nano-clusters and kinetic details of crystallization have been investigated by atomic tracing method^27^,^28^, the details of the atomic-scale structures in metallic glasses, especially, the configuration evolutions and hereditary characteristics for ICO nano-clusters formed in solidification processes of liquid metals, are still not be elucidated.

In this paper, the formation, evolution and heredity characteristics of ICO nano-clusters in a Mg_70_Zn_30_ alloy system consisting of 10^7^ atoms during rapid solidification, have been studied in detail by classic molecular dynamics (MD) simulation. The interatomic potentials of Mg_70_Zn_30_ alloy used here are the effective pair potential that is derived from the generalized nonlocal model pseudo-potential (GNMP) based upon the first-principle interaction force in the second order perturbation theory, which were defined in detail in Refs 29 and 30. The microstructures are analyzed by Honeycutt-Anderson (HA) bond-type index method^31^ and cluster-type index method (CTIM)^32^. The more complicated configurations of ICO nano-clusters have been clearly displayed and some new interesting results obtained as follows.

## Results

### Pair distribution function analysis

**Figure 1:**
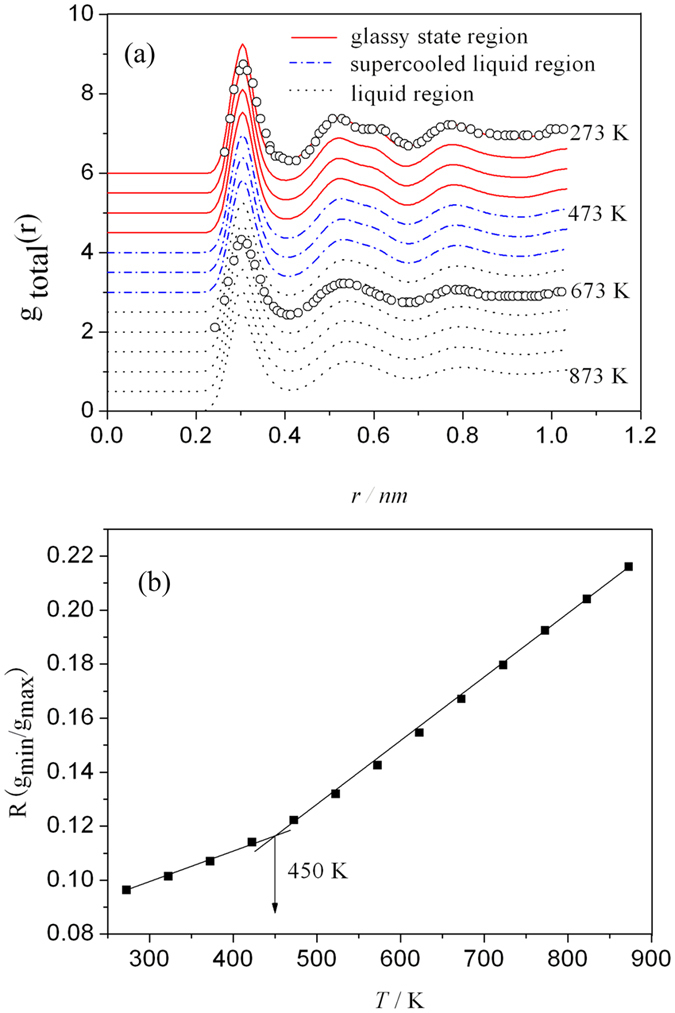
(**a**) Pair distribution function g(r) curves at different temperatures during the cooling process of liquid Mg_70_Zn_30_ alloy. Experimental points are taken from ref. 33 measured by neutron diffraction. (**b**) Relations of Wendt-Abraham ratio *R* = *g*_*min*_/*g*_*max*_ with temperature obtained from (**a**). where *g*_*min*_ and *g*_*max*_ are the magnitudes of the first valley and first peak on a PDF curve.

Since the PDF (g(r)) is a Fourier transformation of the structure factor S(q) obtained from diffraction experiment, it can be used to verify the simulation results. Figure 1(a) displays the PDF curves at different temperatures during the cooling process of liquid Mg_70_Zn_30_ alloy, in which the experimental results of the PDFs at 673 K (liquid) and 293 K (glass) come from a conversion of the reduced PDF in ref. 33 measured by neutron diffraction. From Fig. 1(a), it is clearly seen that the splitting of the second peak of the total PDF becomes pronounced with the decreasing temperature, which indicates the formation of Mg_70_Zn_30_ metallic glass and the enhancement of SRO during the quenching processes. The total PDFs for the liquid and glass structures both agree well with the experimental results. Based on the simulated total PDF, the glass transition temperature *Tg* is estimated to be 450 K by the relation of the Wendt-Abraham ratio *R*^34^ with temperature, as shown in Fig. 1(b).

### The formation of IS-ICO clusters

**Figure 2:**
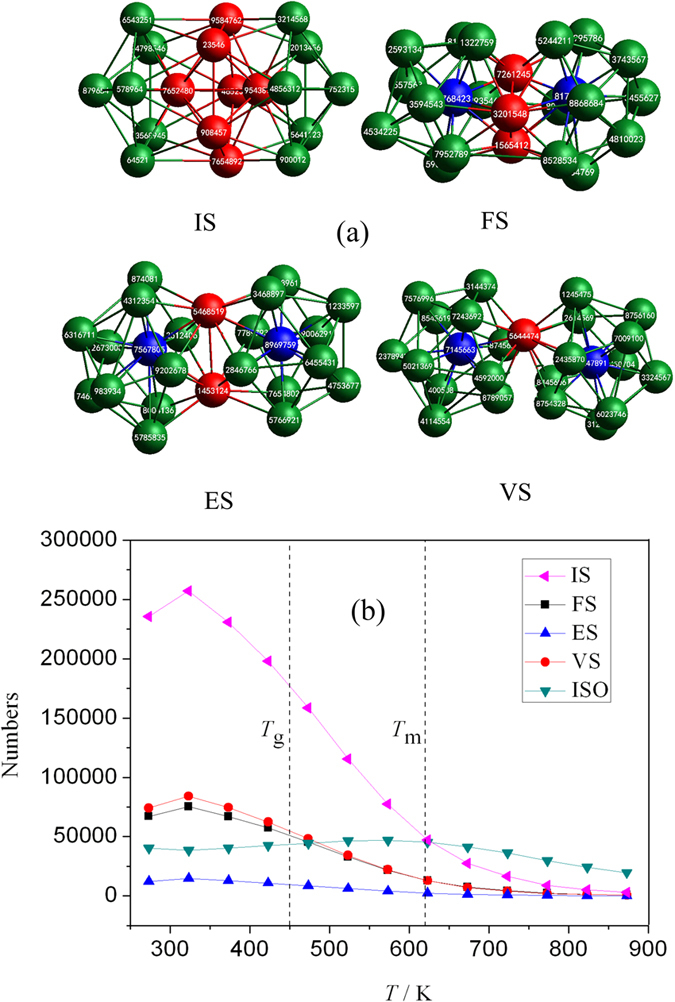
(**a**) Schematics for four linkages of two ICOs by sharing 7, 3, 2 and 1 atoms with each other in turn: intercross-sharing (IS), face-sharing (FS), edge-sharing (ES), and vertex-sharing (VS) (For clarity, all shared atoms are in red); (**b**) Evolutions of the numbers for IS, FS, ES, VS nano-clusters and ISO (isolated icosahedron) in the system with temperature.

The simplest ICO nano-cluster is consisting of 2 ICOs (namely 2 ICO basic clusters). The schematics for four linkages of 2 ICOs are shown in Fig. 2(a). It can be clearly seen that 2 ICOs can be combined into four types of ICO nano-clusters by sharing 7, 3, 2, and 1 atoms (as shown with red color in Fig. 2(a)) with each other in turn. In other word, there are 4 sharing modes of intercross-sharing (IS), face-sharing (FS), edge-sharing (ES), and vertex-sharing (VS) for 2 ICOs, containing 19, 23, 24 and 25 atoms, respectively^35^. For short, the combination of 2 ICOs by IS linkage is called as an IS-ICO cluster (nano-cluster). Particularly, with 7 sharing atoms, the IS-ICO clusters would be more tightness and stable than other three linkages.

Figure 2(b) shows the evolutions of the numbers for IS, FS, ES, VS nano-clusters and ISO (isolated icosahedron with 13 atoms) (for comparison) in the system with temperature. From this, it can be clearly seen that as *T* > *T*_m_, most of icosahedra are isolated, only few linked ICO-clusters exist in the system. At *T* < *T*_m_, the isolated ICO and ES clusters almost have no obvious changes, while other three linkage ICO-clusters are increased. Especially, only the number of IS-ICO-cluster rapidly increased and much higher than others, which will be further investigated in depth below.

### The magic number sequence of IS-ICO clusters

**Figure 3:**
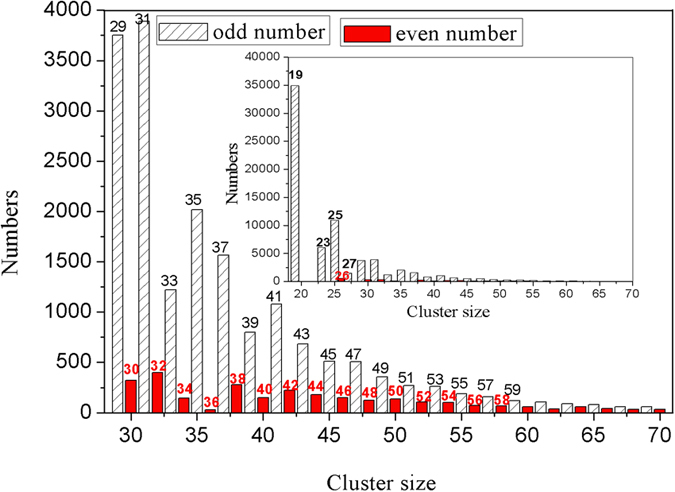
Probability distribution of IS-ICO clusters in Mg_70_Zn_30_ metallic glass at 273 K. Inset is the magic number spectra in the range of 19–70 (atom number). Sparse-gray-line box for odd number; solid red box for even number.

**Figure 4:**
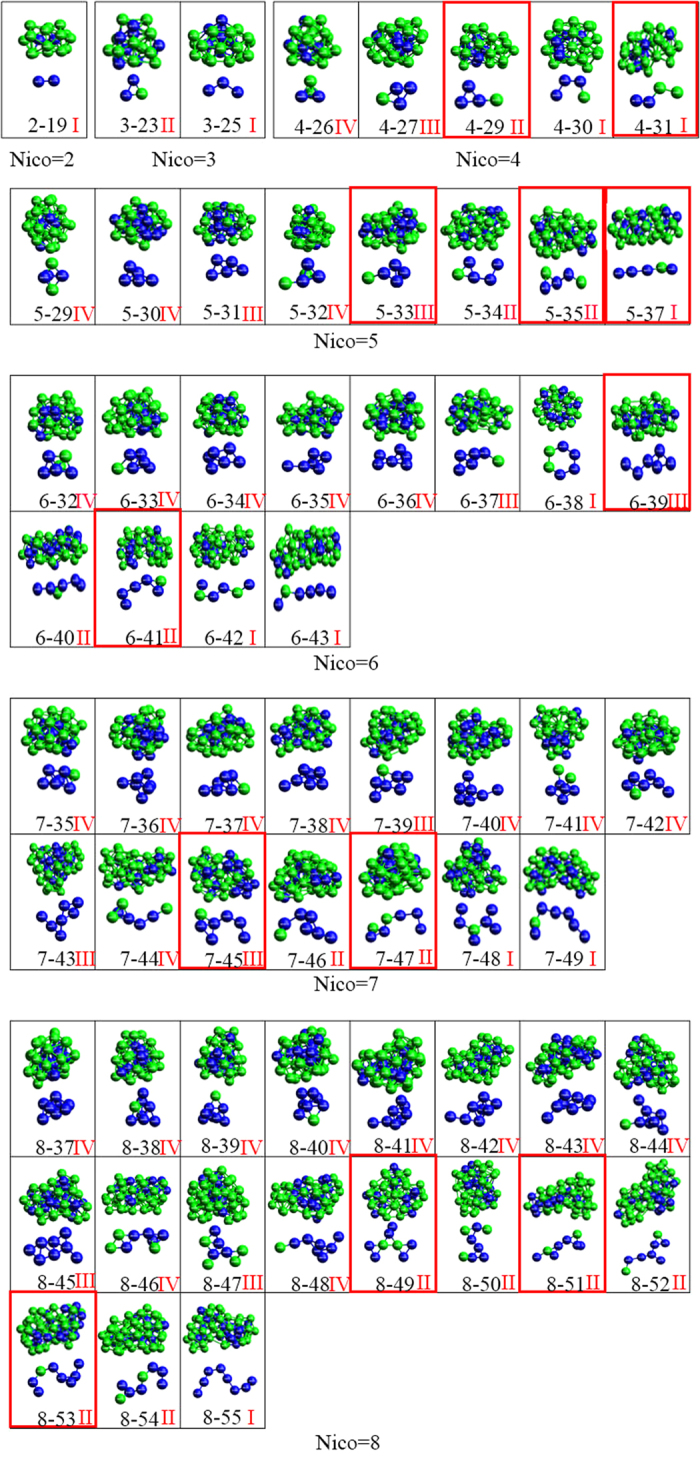
The geometries of IS-ICO clusters composed of 2-8 icosahedra in Mg_70_Zn_30_ metallic glass at 273 K. Each box is for a kind of *i-N* IS-ICO clusters: the upper pictures is the whole cluster; the lower is the configuration of central atoms; and the red Roma number at the right-bottom is the linkage type. Those in red boxes have higher probability. Green is Mg atoms and blue is Zn atoms.

The atom number distribution of various IS-ICO clusters in the system at 273 K as shown in Fig. 3. It can be found that almost all the IS-ICO clusters with possible sizes (by the atom number *N)* can exist in the system. Highly interesting, the relative probabilities of IS-ICO clusters with odd atom number *N* are always far higher than those with even atom number. It may be connected with geometries or energy of each cluster^26^. Thus we will mainly discuss below the odd magic number sequence of 19, 23, 25, 27, 29, 31, 33, 35, 37, 39, 41, 43, 45, 47, 49, 51, 53, 55 … and so on. There is only one geometrical configuration for five types of IS-ICO clusters with *N* = 19, 23, 25, 26 and 27, respectively; while for others (*N* >= 29) there are at least two geometrical configurations. Thus each type of IS-ICO cluster is assigned an ID of *i*-*N* (such as 4–29) as shown in Fig. 4, where *i* is the number of ICOs, and *N* is the number of involved atoms.

### The geometries of IS-ICO clusters

The geometrical configurations of all the IS-ICO clusters including 2~8 IS-ICOs in the system at 273 K are shown in Fig. 4. According to the configurations of central atoms in the IS-ICO clusters, all the IS-ICO clusters can be classified into four types: (I) Chain-like, in which all the central atoms of IS-ICO clusters form a single open chain (central-atom-chain). Eleven types of IS-ICO clusters of 2–19, 3–25, 4–30, 4–31, 5–37, 6–38, 6–42, 6–43, 7–48, 7–49 and 8–55 display this feature. The total number of chain-like IS-ICO clusters is 51448 in the system; (II) Triangle-tailed, in which 3 central atoms form a triangle and they linked into one or two central-atom-chains. Fourteen types of IS-ICO clusters of 3–23, 4–29, 5–34, 5–35, 6–40, 6–41, 7–44, 7–46, 7–47, 8–49, 8–50, 8–51, 8–52, 8–53 and 8–54 display this feature. The total number of triangle-tailed IS-ICO clusters is 13470; (III) Quadrilateral-tailed, in which 4 central atoms form a quadrilateral and they linked into one or two central-atom-chains. Ten types of IS-ICO clusters of 4–27, 5–31, 5–33, 6–37, 6–39, 7–39, 7–43, 7–45, 8–45 and 8–47 display this feature. The total number of quadrilateral-tailed IS-ICO clusters is 5089; (IV) Tetrahedron-tailed, in which 4 centres form a tetrahedron and they linked into one or two central-atom-chains. Fifteen types of IS-ICO clusters of 4–26, 5–29, 5–30, 5–32, 6–32, 6–33, 6–34, 6–35, 6–36, 7-35, 7–36, 7–37, 7–38, 7–40, 7–41, 7–42, 7–44, 8–37, 8–38, 8–39, 8–40, 8–41, 8–42, 8–43, 8–44, 8–46 and 8–48 display this feature. The total number of tetrahedron-tailed IS-ICO clusters is 3016.

From the aforementioned it can be clearly found that the IS-ICO clusters with different central atom configurations would be corresponding to different stacking densities. The Chain-like (I) IS-ICO clusters is the most loose, the Tetrahedron-tailed (IV) is the most tight, and the Triangle-tailed (II) and Quadrilateral-tailed (III) are in the middle.

Going further, based on the total numbers of the each type of IS-ICO clusters in the system being in the order of 51448(I), 13470(II), 5089(III) and 3016(IV), it can be clearly seen that the more loose the IS-ICO cluster is, the greater the number it appear in the system.

**Figure 5:**
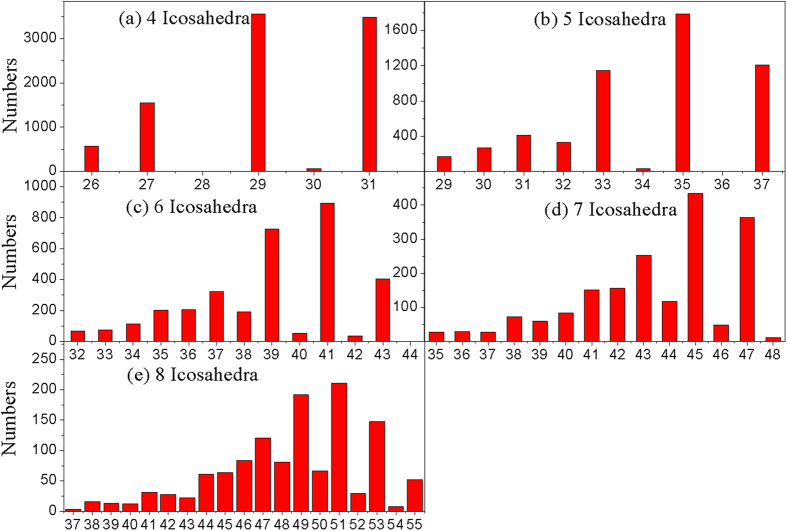
The number of IS-ICO clusters composed of 4, 5, 6, 7, 8 icosahedra in Mg_70_Zn_30_ metallic glass at 273 K. The IS-ICO clusters with higher probability are as for (**a**) the 4–29 and 4–31 IS-ICO clusters, (**b**) the 5–33, 5–35 and 5–37 IS-ICO clusters, (**c**) the 6–39 and 6–41 IS-ICO clusters, (**d**) the 7–45 and 7–47 IS-ICO clusters, and (**e**) the 8–49, 8–51 and 8–53 IS-ICO clusters, respectively.

For deep understanding the special distributions of various nano-clusters in the system, the numbers of IS-ICO clusters composed of 4, 5, 6, 7 and 8 icosahedra at 273 K are shown in Fig. 5 in turn. It can be found that the numbers of 4–29 and 4–31 IS-ICO clusters are larger among the 5 4-N IS-ICO clusters; the 5–33, 5–35 and 5–37 IS-ICO clusters are larger among the 8 5-N IS-ICO clusters; the 6–39 and 6–41 IS-ICO clusters are larger among the 12 6-N IS-ICO clusters; the 7–43, 7–45 and 7–47 IS-ICO clusters are larger among the 15 7-N IS-ICO clusters; the 8–49, 8–51 and 8–53 IS-ICO clusters are larger among the 19 8-N IS-ICO clusters, respectively. Furthermore, among the IS-ICO clusters composed of the same number of ICOs, the clusters with higher probability usually have larger sizes. For instance, the numbers of chain-like (4–31), triangle-tailed (4–29), quadrilateral- tailed (4–27) and tetrahedrontailed (4–26) 4-N IS-ICO clusters, are 3536, 3559, 1552 and 574 respectively, as shown in Fig. 4 (Nico = 4) and Fig. 5(a).

It should be pointed out that those clusters with the highest probability are not necessary to have the largest sizes. The clusters with the highest probability are usually in the second place of the larger sizes. On the other hand, it also can be clearly seen that the clusters with higher packing density, such as the quadrilateral-tailed(III) and tetrahedron-tailed(IV), have more structure-types but their amount is less, and those with lower packing density, such as the chain-like(I) and triangle-tailed(II), have more amount, but their types is less. For example, the tetrahedron-tailed(IV) 8-N IS-ICO has ten structure-types but only 28.26% of the total number for all 8-N IS-ICO clusters.

### The heredity of IS-ICO clusters

In order to deeply understand the critical role of icosahedra and the formation mechanism of the IS-ICO nano-clusters during the rapid solidification of Mg_70_Zn_30_ alloy, the heredity and evolution of IS-ICO clusters are further investigated by means of an inverse tracking method. For convenience of discussion, in this work, the heredity of basic clusters is defined as follows. When temperature decreases from *T*_*1*_ to *T*_*2*_ (*T*_*2*_ < *T*_*1*_), if a 13-atom icosahedron does not changed at all, it is a perfect heredity; if only the central atom and part of the 12 neighbors keep unchanged, it is a partial (or replacement) heredity, i.e., the core heredity, and distinguished by the number of changed atoms. For example, if 4 neighboring atoms are changed, it is called as a 4-replacement heredity, and so on.

We adopted *f*_*i*_ to express the descendible fraction of *i*-*N* ICO cluster in the system. When temperature decreases from *T*_1_ to *T*_2_ (*T*_*2*_ < *T*_*1*_), if *x* of *N* ICOs are unchanged, the descendible fraction for perfect heredity is defined as *f*_*ip*_* = *(*x*/*N*) × 100%; and if *y* of *N* ICOs take 4-replacement heredity, then descendible fraction for 4-replacement heredity is *f*_*ir*_ = (*y*/*N*) × 100%; obviously (*f*_*ip*_* + Σf*_*ir*_) ≤ 100% at any temperature.

**Figure 6:**
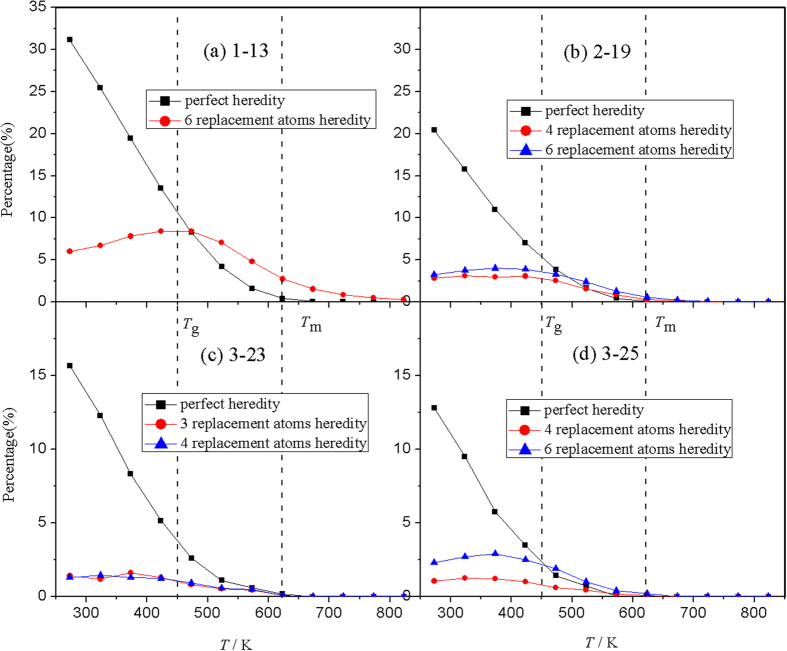
The descendible fractions of four IS-ICO nano-clusters during the cooling process. (**a**) The perfect and 6-replacement heredity for the 13-atom ICOs. (**b**) The perfect heredity and 4-, 6-replacement heredity for 2–19 IS-ICO clusters. (**c**) The perfect heredity and 3-, 4-replacement heredity for 3–23 IS-ICO clusters. (**d**) The perfect heredity and 4-, 6-replacement heredity for 3–25 IS-ICO clusters.

The temperature dependence of descendible fraction for perfect heredity (*f*_*ip*_) and replacement heredity (*f*_*ir*_) are shown in Fig. 6. Figure 6(a) clearly reveals that for the single icosahedra (i.e. 1–13 cluster), at *T* > *T*_*m*_ there is no perfect heredity (*f*_*ip*_) and only some 6-replacement heredity (*f*_*ir*_) can be found; at the super-cooled liquid state, the perfect heredity emerges at *T*_*m*_ and then increases rapidly, and the 6-replacement heredity has a significant ascent and gets the maximum at around *T*_*g*_; at *T* < *T*_*g*_ the perfect heredity keeps the rapid increase, while the 6-replacement heredity decreases gently. For the other three (2–19, 3–23 and 3–25) IS-ICO clusters depicted in Fig. 6(b–d), the evolution of the perfect heredity is similar to that for the 1–13 cluster; while the last value of the perfect heredity decreases with the increase of the IS-ICO cluster. The major replacement heredities for the 2–19, 3–23 and 3–25 IS-ICO clusters are different. The 4- and 6-replacement heredities are for the 2–19 and 3–25 IS-ICO clusters; while 3-and 4-replacement heredities are for the 3–23 IS-ICO clusters. The x-replacement heredities (*f*_*ir*_) reach the maximum value at a temperature slightly below *T*_*g*_ and then decrease gently.

### The replacement heredity modes of IS-ICO clusters

**Figure 7:**
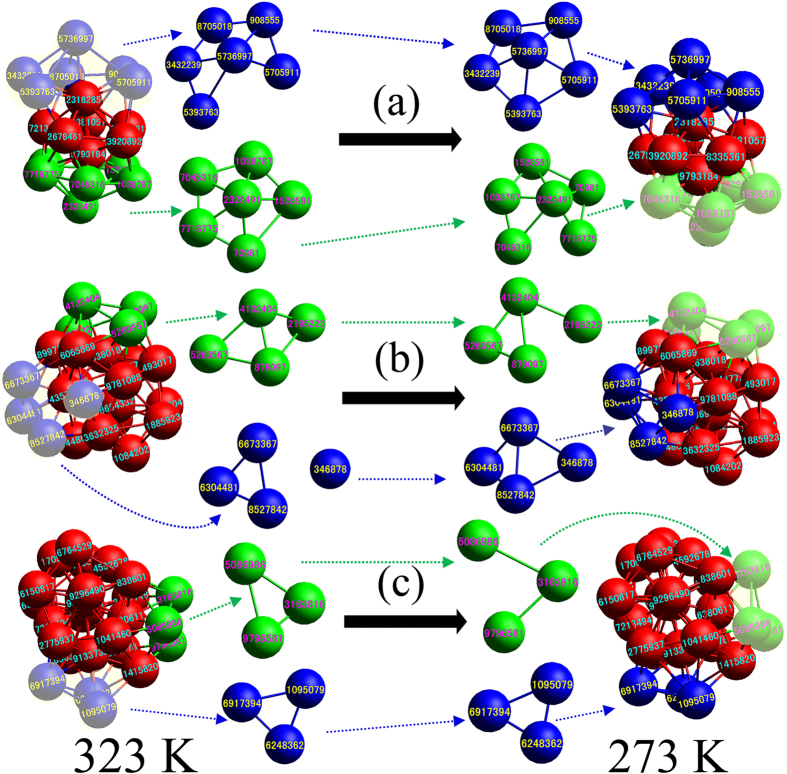
The visualization of replacement heredity between 323 K and 273 K. (**a**) 6 replacement heredity for the 1–13 cluster (13-atom ICO); (**b**) 4 replacement heredity for the 2–19 IS-ICO cluster; (**c**) 3 replacement heredity for the 3–23 IS-ICO cluster. Red atoms stay unchanged; green atoms fall away from red atoms; and blue atoms recombine with red atoms.

For the single icosahedron (1–13 cluster), the pentagonal pyramid composed of 6 atoms usually is replaced as whole as shown in Fig. 7(a). It can be clearly seen that the icosahedron can be considered to be composed of a pentagonal pyramid (6 green atoms) and a pentagonal bipyramid (7 red atoms). Near the icosahedron another 6 blue atoms do not comprises a pentagonal pyramid at 323 K. With the decrease of temperature, the 6 blue atoms comprise a pentagonal pyramid that takes place the previous pentagonal pyramid formed of 6 green atoms and reconstitutes a new icosahedra together with the 7-atom pentagonal bipyramid existed at 273 K. By the similar way, Fig. 7(b) and (c) illustrates that the 4- and 3-replacement heredity for the 2–19 and 3–23 IS-ICO clusters respectively. The aforementioned analysis reveals the replacement heredity usually conducted via local structures composed of a group of atoms (such as 3, 4, 6 atoms) rather than individual atoms.

### The perfect heredity fraction in IS-ICO nano-clusters

**Figure 8:**
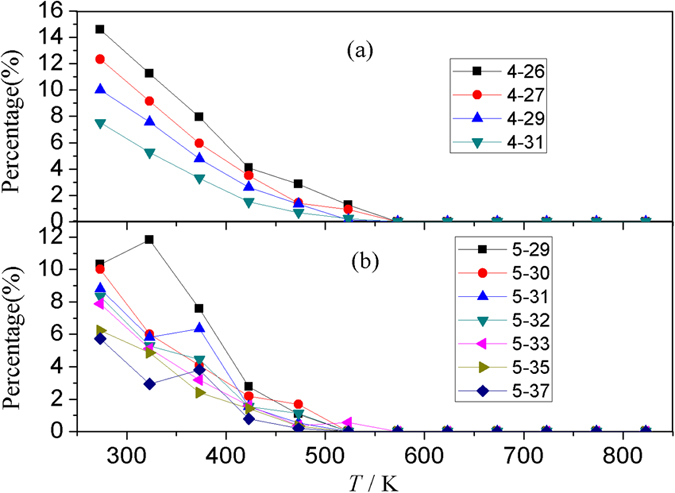
The perfect heredity fraction of IS-ICO nano-clusters during the cooling process. (**a**) For the 4-N h-ICO clusters. (**b**) For the 5-N IS-ICO clusters.

The evolution of the perfect heredity with temperature for 4-N and 5-N IS-ICO clusters is shown in Fig. 8. It can be clearly seen that for the IS-ICO clusters composed of the same number of ICOs, the smaller the atom number in the IS-ICO cluster, the higher the perfect heredity.

Generally, for a group of IS-ICO clusters that have the same number of ICOs, the smaller the atom number in the IS-ICO cluster, the higher the perfect heredity and the more compact the configuration of atoms; and in the ascending order of the atom number in a IS-ICO cluster, they correspond to the pyramid-tailed, quadrilateral-tailed, triangle-tailed, and chain-like IS-ICO clusters, respectively. Therefore, the more compact the interconnection between ICOs in an IS-ICO cluster, the more stable it is.

## Discussion

In this paper we found that the IS-ICO clusters exist in the larger system with different probabilities, and the numbers of IS-ICO clusters with high-probability demonstrate an odd magic number sequence in turn as 19, 23, 25, 27, 29, 31, 33, 35, 37, 39, 41, 43, 45, 47, 49, 51,53, 55 … and so on. For all the IS-ICO clusters including 2~8 ICOs, there are 62 kinds of geometrical structures in the system. The nano-clusters can be classified into four types: chain-like, triangle-tailed, quadrilateral-tailed and pyramidal-tailed, classified by the geometrical structure of the central atoms in such clusters.

And among a group of IS-ICO clusters that have the same number of ICOs, the clusters with high-probability usually are the larger size ones. For the IS-ICO nano-clusters formed of 1–3 icosahedra, the perfect heredity emerges when temperature is slightly less than *T*_*m*_, and a significant ascent of *f*_*ip*_ at *T*_*m*_ > *T* > *T*_*g*_ makes the perfect heredity exceeded replacement heredity *f*_*ir*_ at around *T*_*g*_. The replacement heredity mainly takes place with 6-, 4-, and 3-atom replacement, corresponding to the pentagonal pyramid, quadrangle, and triangle local structures, rather than individual atoms. For the IS-ICO clusters formed of the same number icosahedra, the lower the atom number, the higher the perfect heredity, the more compact the configuration of atoms and the more stable they are.

## Methods

### MD simulations

The MD technique and simulation conditions used in this work are as follows. At first, let the 10^7^ atoms (i.e., 7* *×* *10^6 ^Mg atoms and 3* *×* *10^6^ Zn atoms) of Mg_70_Zn_30_ alloy place in a cubic box. The cubic box size is determined by both the number of atoms in the system and the mean volume of each atom at each given temperature under constant pressure. The simulation is performed in NVT ensemble, under periodic boundary conditions. The interatomic potentials of Mg_70_Zn_30_ alloy used here are the effective pair potential that is derived from the generalized nonlocal model pseudo-potential (GNMP) based upon the first-principle interaction force in the second order perturbation theory, which were defined in detail in refs 29 and 30. The pair-wise potentials are cut off at 20 a.u. (atomic unit). The time step is 5 × 10^−15^ s. For the simple metals and their alloys, the accuracy and reliability of this potential have been extensively demonstrated by computing their structural, dynamic, and thermodynamics properties^34^,^35^,^36^. First of all, this simulation calculation is started at 873 K (the melting point *T*_m_ of Mg_70_Zn_30_ alloy is about 616 K), let the system run 20000 time steps to obtain a well equilibrated liquid state. The damped force method^37^,^38^,^39^ is adopted to control the temperature of the system by decreasing linearly from 873 to 273 K at cooling rate of 1 × 10^13^ K/s. At each given temperature, the system is relaxed for 400 time steps, and then the instantaneous spatial coordinates of each atom as well as other parameters are recorded for further analysis.

### Inverse tracking method

In this paper, as the same number of atoms and geometry for IS-ICO clusters formed of 1–5 icosahedra are traced from temperature *T*_1_ to temperature *T*_2_. The influence of temperature on the heredity fraction of IS-ICO clusters are also investigated by the following equation: *f*_*i*_ = (*N*_T1_/*N*_T2_) × 100%, where *N*_T1_ is the number of inheritable *i-N* IS-ICO clusters at *T*_*1*_. *N*_T2_ is the total number of *i-N* IS-ICO clusters at *T*_*2*_(<*T*_*1*_)^27^,^28^.

### Cluster type index method

**Figure 9:**
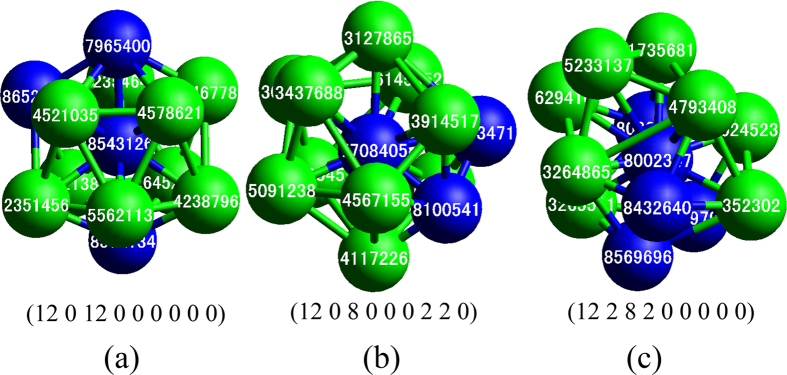
Three basic clusters in Mg_70_Zn_30_ system. (**a**) An icosahedron (12 0 12 0 0 0 0 0 0) with center atom of 8543126; (**b**) A defective icosahedron (12 0 8 0 0 0 2 2 0) with central atom of 7708405; (**c**) A defective icosahedron (12 2 8 2 0 0 0 0 0) with central atom 8002347. (Green is Mg atoms and blue is Zn atoms).

In order to concisely characterize clusters in a system, based on the work of Qi and Wang^40^, the authors have proposed a cluster-type index method (CTIM), as shown in detail in ref. 30. In CTIM, a basic cluster is defined as composed of a center atom and its surrounding atoms. At present, for deep understanding the specific structure characteristics of this system, based on our previous works, a new cluster-type index method (CTIM-3) have been proposed. In CTIM-3, a basic cluster is expressed by nine (integer) index of (*N, n*_1_, *n*_2_, *n*_3_, *n*_4_, *n*_5_, *n*_6_, *n*_7_, *n*_8_), where *N* is the number of surrounding atoms of the central atom in the basic cluster, namely, the coordination number(CN), and *n*_1_, *n*_2_, *n*_3_, *n*_4_, *n*_5_, *n*_6_, *n*_7_, *n*_8_ in turn denote the numbers of 1441, 1551, 1661, 1421, 1422, 1541, 1431 and 1321 bond-types (expressed by Honeycutt-Anderson (HA) bond-type index method^31^) connected surrounding atoms to the central atom. For example, the icosahedron basic cluster can be expressed by (12 0 12 0 0 0 0 0 0); similarly, the BCC, FCC, HCP basic clusters expressed in turn by (14 6 0 8 0 0 0 0 0), (12 0 0 0 12 0 0 0 0) and (12 0 0 0 6 6 0 0 0) respectively^41^. Figure 9 shows an icosahedron (ICO) and two defective ICOs selected in the Mg_70_Zn_30_ system.

**Figure 10:**
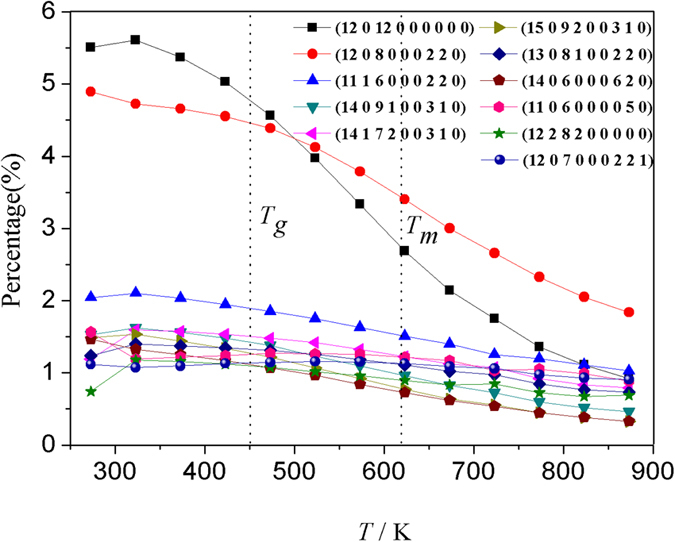
Relations of the percentage of major basic clusters with temperature.

At 273 K, there are totally 8502 kinds of basic clusters that can be described by CTIM-3 as shown in Fig. 10. It can be clearly seen that for the top 11 basic clusters, the most prominent increase occurs in the supercooled liquid region (*T*_m_ ~ *T*_g_). With decreasing temperature, the ICO basic cluster (12 0 12 0 0 0 0 0 0) and defect ICO basic cluster (12 0 8 0 0 0 2 2 0) increase remarkably at *T* < *T*_m_, while others are only slightly increased. This demonstrates that the two basic clusters play a critical role in the microstructure transition during rapid solidification processes.

## Additional Information

**How to cite this article:** Liang, Y.-C. *et al*. Structural evolutions and hereditary characteristics of icosahedral nano-clusters formed in Mg_70_Zn_30_ alloys during rapid solidification processes. *Sci. Rep.* **7**, 43111; doi: 10.1038/srep43111 (2017).

**Publisher's note:** Springer Nature remains neutral with regard to jurisdictional claims in published maps and institutional affiliations.

## References

[b1] Shechtman D., Blech I., Gratias D. & Cahn J. W. Metallic Phase with Long-Range Orientational Order and No Translational Symmetry. Phys. Rev. Lett. 53, 1951–1954 (1984).

[b2] Sauda J., Matsushita M. & Inoue A. Direct observation of icosahedral cluster in Zr_70_Pd_30_ binary glassy alloy. Appl. Phys. Lett. 79, 412–414 (2001).

[b3] Liu A. C. Y. . Systematic Mapping of Icosahedral Short-Range Order in a Melt-Spun Zr_36_Cu_64_ Metallic Glass. Phys. Rev. Lett. 110, 205505–P5 (2013). 25167428 10.1103/PhysRevLett.110.205505

[b4] Zhang Y., Mattern N. & Eckert J. Atomic structure and transport properties of Cu_50_Zr_45_Al_5_ metallic liquids and glasses: Molecular dynamics simulations. J. Appl. Phys. 110, 093506-P8 (2011).

[b5] Ding J., Cheng Y. Q., Sheng H. & Ma E. Short-range structural signature of excess specific heat and fragility of metallic-glass-forming supercooled liquids. Phys. Rev. B. 85, 060201–P5 (2012).

[b6] Hao S. G., Wang C. Z., Li M. Z., Napolitano R. E. & Ho K. M. Dynamic arrest and glass formation induced by self-aggregation of icosahedral clusters in Zr_1−x_Cu_x_ alloys. Phys. Rev. B. 84, 064203–P4 (2011).

[b7] Wang H., Hu T., Qin J. Y. & Zhang T. Local structure origin of higher glass forming ability in Ta doped Co_65_B_35_ amorphous alloy. J. Appl. Phys. 112, 073520–P5 (2012).

[b8] Cheng Y. Q. & Ma E. Indicators of internal structural states for metallic glasses: Local order, free volume, and configurational potential energy. Appl. Phys. Lett. 93, 051910–P3 (2008).

[b9] Bochicchio D. & Ferrando R. Morphological instability of core-shell metallic nanoparticles. Phys. Rev. B 87, 165435–P13 (2013).

[b10] Ferrando R. Symmetry breaking and morphological instabilities in core-shell metallic nanoparticles. J. Phys.: Condens. Matter 27, 013003–P35 (2015). 25485754 10.1088/0953-8984/27/1/013003

[b11] Rossi G. . Magic Polyicosahedral Core-Shell Clusters. Phys. Rev. Lett. 93, 105503–P4 (2004). 15447416 10.1103/PhysRevLett.93.105503

[b12] Barcaro G., Ferrando R., Fortunelli A. & Rossi G. Exotic Supported CoPt Nanostructures: From Clusters to Wires. J. Phys. Chem. Lett. 1, 111–115 (2010).

[b13] Mechler S., Schumacher G., Zizak I., Macht M. P. & Wanderka N. Correlation between icosahedral short range order, glass forming ability, and thermal stability of Zr-Ti-Ni-Cu-(Be) glasses. Appl. Phys. Lett. 91, 021907–P3 (2007).

[b14] Cheng Y. Q., Ma E. & Sheng H. W. Atomic level structure in multicomponent bulk metallic glass. Phys. Rev. Lett. 102, 245501–245504 (2009). 19659024 10.1103/PhysRevLett.102.245501

[b15] Cheng Y. Q. & Ma E. Atomic-level structure and structure–property relationship in metallic glasses. Prog. Mater. Sci. 56, 379–473 (2011).

[b16] Lee M., Lee C. M., Lee K. R., Mad E. & Lee J. C. Networked interpenetrating connections of icosahedra: Effects on shear transformations in metallic glass. Acta Mater. 59, 159–170 (2011).

[b17] Wakeda M. & Shibutani Y. Icosahedral clustering with medium-range order and local elastic properties of amorphous metals. Acta Mater. 58, 3963–3969 (2010).

[b18] Lad K. N., Jakse N. & Pasturel A. Signatures of fragile-to-strong transition in a binary metallic glass-forming liquid. J. Chem. Phys. 136, 104509–P8 (2012). 22423850 10.1063/1.3692610

[b19] Li M. Z., Wang C., Hao S., Kramer M. & Ho K. Structural heterogeneity and medium-range order in Zr_x_Cu_100−x_ metallic glasses. Phys. Rev. B. 80, 184201–P7 (2009).

[b20] Fang H. Z., Hui X., Chen G. L. & Liu Z. K. Al-centered icosahedral ordering in Cu_46_Zr_46_Al_8_ bulk metallic glass. Appl. Phys. Lett. 94, 091904–P3 (2009).

[b21] Huang L. . Medium-range icosahedral order in quasicrystal-forming Zr_2_Pd binary metallic glass. Appl. Phys. Lett. 98, 231906–P3 (2011).

[b22] Hui X. . Icosahedral ordering in Zr_41_Ti_14_Cu_12.5_Ni_10_Be_22.5_ bulk metallic glass. Appl. Phys. Lett. 92, 201913–P3 (2008).

[b23] Pawlak R. . Chain-like structure elements in Ni_40_Ta_60_ metallic glasses observed by scanning tunneling microscopy. Sci. Rep. 5, 1314 (2015). 10.1038/srep13143 PMC4542518 26268430

[b24] Liu R. S. . Formation and magic number characteristics of clusters formed during solidification processes. J. Phys.: Condens. Matter 19, 196103–P17 (2007).

[b25] Liu R. S. . Simulation study of size distributions and magic number sequences of clusters during the solidification process in liquid metal Na. J. Non-Cryst. Solids 355, 541–547 (2009).

[b26] Doye J. P. K., Wales D. J., Zetterling F. H. M. & Dzugutov M. The favored cluster structures of model glass formers. J. Chem. Phys. 118, 2792–2799 (2003).

[b27] Hou Z. Y. . Simulation study on the formation and evolution properties of nano-clusters in rapid solidification structures of sodium. Modell. Simul. Mater. Sci. Eng. 15, 911–922 (2007).

[b28] Wen D. D., Peng P., Jiang Y. Q. & Liu R. S. On the heredity and evolution of icosahedral clusters during the rapid solidification of liquid Cu_50_Zr_50_ alloys. J. Non.-Cryst. Solids. 378, 61–70 (2013).

[b29] Wang S. & Lai S. K. Structure and electrical resistivity of liquid binary alloys. J. Phys. F. 10, 2717–2737 (1980).

[b30] Li D. H., Li X. R. & Wang S. Variational calculation of Helmholtz free energies with applications to the sp-type liquid merals. J. Phys. F. 16, 309–321 (1986).

[b31] Honeycutt J. D. & Anderson H. C. Molecular-dynamics study of melting and freezing of small Lennard-Jones clusters. J. Phys. Chem. 91, 4950–4963 (1987).

[b32] Liu R. S., Dong K. J., Li J. Y., Yu A. B. & Zou R. P. Formation and description of nano-clusters formed during rapid solidification process of liquid metals. J. Non.-Cryst. Solids. 351, 612–617 (2005).

[b33] Andonov P. & Chieux P. Structural study of eutectic Mg_0.72_Zn_0.28_ alloy: I. Local order in the amorphous and liquid states Comparison with the crystalline phase Mg_51_Zn_20_. J. Non.-Cryst. Solids. 93, 331–349 (1987).

[b34] Wendt H. R. & Abraham F. F. Empirical criterion for the glass transition region based on Monte Carlo simulations. Phys. Rev. Lett. 41, 1244–1246 (1978).

[b35] Liang Y. C. . Influence of icosahedral order on the second peak splitting of pair distribution function for Mg70Zn30 metallic glass. J. Alloys Compd. 597, 269–274 (2014).

[b36] Jin Z. H., Lu K., Gong Y. D. & Hu Z. Q. Glass transition and atomic structures in supercooled Ga_0.15_Zn_0.15_Mg_0.7_ metallic liquids: A constant pressure molecular dynamics study. J. Chem. Phys. 106, 8830–8840 (1997).

[b37] Hoover W. G., Ladd A. J. C. & Moran B. High-strain-rate plastic flow studied via nonequilibrium molecular dynamics. Phys. Rev. Lett. 48, 1818–1820 (1982).

[b38] Evans D. J. Computer “experiment” for nonlinear thermodynamics of Couette flow. J. Chem. Phys. 78, 3297–3302 (1983).

[b39] Brown D. & Clarke J. H. R. A comparison of constant energy, constant temperature and constant pressure ensembles in molecular dynamics simulations of atomic liquids. Mol. Phys. 51, 1243–1252 (1984).

[b40] Qi D. W. & Wang S. Icosahedral order and defects in metallic liquids and glasses. Phys. Rev. B 44, 884–887 (1991). 10.1103/physrevb.44.884 9999205

[b41] Zhou L. L. . Crystallization characteristics in supercooled liquid zinc during isothermal relaxation: A molecular dynamics simulation study. Sci. Rep. 6, 31653 (2016). 27526660 10.1038/srep31653 PMC4985810

